# Antibody-Specific Model of Amino Acid Substitution for Immunological Inferences from Alignments of Antibody Sequences

**DOI:** 10.1093/molbev/msu340

**Published:** 2014-12-21

**Authors:** Alexander Mirsky, Linda Kazandjian, Maria Anisimova

**Affiliations:** ^1^Systems Biophysics and Functional Nanosystems, Ludwig-Maximilians-Universität München, München, Germany; ^2^Department of Computer Science, Swiss Federal Institute of Technology (ETH Zürich), Zürich, Switzerland; ^3^MAB Discovery GmbH, Neuried, Germany; ^4^Institute of Applied Simulation (IAS), School of Life Sciences and Facility Management, Zürich University of Applied Sciences (ZHAW), Wädenswil, Switzerland

**Keywords:** Markov model, amino acid substitution, alignment, evolution, antibody, somatic hypermutation, antibody genealogy

## Abstract

Antibodies are glycoproteins produced by the immune system as a dynamically adaptive line of defense against invading pathogens. Very elegant and specific mutational mechanisms allow B lymphocytes to produce a large and diversified repertoire of antibodies, which is modified and enhanced throughout all adulthood. One of these mechanisms is somatic hypermutation, which stochastically mutates nucleotides in the antibody genes, forming new sequences with different properties and, eventually, higher affinity and selectivity to the pathogenic target. As somatic hypermutation involves fast mutation of antibody sequences, this process can be described using a Markov substitution model of molecular evolution. Here, using large sets of antibody sequences from mice and humans, we infer an empirical amino acid substitution model AB, which is specific to antibody sequences. Compared with existing general amino acid models, we show that the AB model provides significantly better description for the somatic evolution of mice and human antibody sequences, as demonstrated on large next generation sequencing (NGS) antibody data. General amino acid models are reflective of conservation at the protein level due to functional constraints, with most frequent amino acids exchanges taking place between residues with the same or similar physicochemical properties. In contrast, within the variable part of antibody sequences we observed an elevated frequency of exchanges between amino acids with distinct physicochemical properties. This is indicative of a sui generis mutational mechanism, specific to antibody somatic hypermutation. We illustrate this property of antibody sequences by a comparative analysis of the network modularity implied by the AB model and general amino acid substitution models. We recommend using the new model for computational studies of antibody sequence maturation, including inference of alignments and phylogenetic trees describing antibody somatic hypermutation in large NGS data sets. The AB model is implemented in the open-source software CodonPhyML (http://sourceforge.net/projects/codonphyml) and can be downloaded and supplied by the user to ProGraphMSA (http://sourceforge.net/projects/prographmsa) or other alignment and phylogeny reconstruction programs that allow for user-defined substitution models.

## Introduction

Antibodies are glycoproteins that constitute a fundamental part of the humoral adaptive immune response and protect all jawed vertebrates (elasmobranches, teleosts, amphibians, reptiles, birds, and mammals) from invading pathogens, such as bacteria, viruses, and parasitic eukaryotes (Das et al. 2012).

Studying and modeling antibody biology and functionality has therefore important influences in several fields: In fact, understanding the tightly regulated mechanisms that govern B lymphopoiesis and antibody maturation is important for understanding the pathogenesis of diseases where these mechanisms are deregulated, such as certain types of autoimmunity, immunodeficiency, and lymphomas. Additionally, antibodies have been used for decades as blockbuster therapeutic drugs in the pharmaceutical industry, mostly in oncotherapy and inflammatory diseases treatment. Especially in this field, bioinformatics modeling of antibody biology should complement laborious experimental techniques, in order to select and develop lead and clinical candidates with desirable properties. Many of these analyses are carried out on a multiplicity of antibody sequences, which are aligned based on homologous residues. Phylogenetic trees can then be derived from such alignments and used to infer the mutational pathways and properties of individual sequences as well as of complete alignments (Barak et al. 2008; Wu et al. 2011; Zhu et al. 2013). Accurate inference of such phylogenies requires a substitution model representing the mutational process under study.

**Fig. 1.**
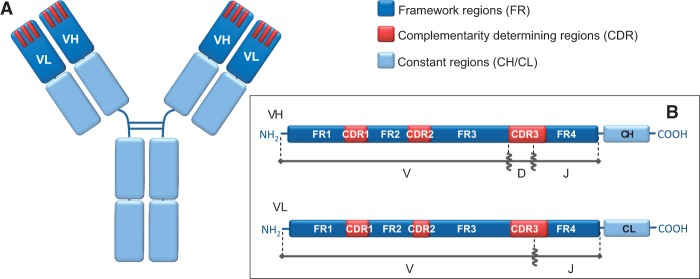
Antibody structure: (*A*) General schematic representation highlighting the apical position of the variable heavy chain (VH) and variable light chain (VL) domains. CDRs are represented as three red bars for each variable region. (*B*) Linearized representation of the variable regions, with their respective original germline gene fragments.

In the last years, antibody research has gained a new momentum thanks to the technological advances in next generation sequencing (NGS), which made it possible to obtain large sequencing data sets at affordable costs and with relatively limited resources (Fischer 2011; Mathonet and Ullman 2013). The availability of such huge data sets allows for, and at the same time demands, the creation of bioinformatics tools for the quantitative analysis of the underlying biological mechanisms. In particular, the availability of large antibody sequencing data can provide an insight into their unique capability to evolve and adapt to new pathogenic targets (antigens) within a few weeks from infection. The surprising plasticity of the antibody repertoire derives from somatic rearrangements and mutational processes taking place in the genome of B lymphocytes, more specifically in the loci encoding for the antibody protein chains (*IgH, IgK*, and *IgL*). These elegant and sophisticated processes are extensively reviewed elsewhere (e.g., Gellert 2002; Chahwan et al. 2012; Xu et al. 2012), and therefore here we only provide a brief overview of such diversification mechanisms. As outlined in figure 1, antibodies can recognize and bind antigens through interactions involving their N-terminal domains, called V (variable) regions, or more precisely, VH for the heavy chain and VL for the light chain. Functional VH and VL regions are assembled in B cell progenitors by piecing together different gene fragments, called V (variability), D (diversity, exists only in the heavy chain loci) and J (joining), chosen from a pool of V, D, and J germlines. This process, known as V(D)J recombination, accounts for most of the combinatorial diversity encountered in antibody repertoires, as germlines belonging to a fragment type show already significant mutual diversity. Furthermore, deletions and insertions of nucleotides at the joining positions between the V, (D), and J fragments considerably increase the number of possible precursor antibodies. Therefore, jawed vertebrates can count on a very flexible system for creating antibody diversity, which in humans can potentially generate up to 10^11^ different antibody precursors (Glanville et al. 2009).

Moreover, additional diversity is introduced later on by a further maturation process, known as somatic hypermutation, which comprises the mutation of nucleotides in the newly created VH and VL region gene segments. This mutational process, coupled with selection of B cells for antigen binding, leads to the optimization of the antibody–antigen interface, resulting in increased binding affinities. It is triggered by the function of activation-induced deaminase, a cellular enzyme that converts cytosines, especially those contained in the specific WRC (W = A/T, R = A/G) sequence motif, to uracils (Rogozin and Kolchanov 1992; Maul and Gearhart 2010). As deoxyuridine (dU) mimics deoxythymidine during replication, the U:G pairing is correctly identified as a mismatch and thus triggers DNA repair mechanisms. In B cells such repair mechanisms are error-prone, so that dU is not always faithfully repaired, thus resulting in point mutations upon cell division (Chahwan et al. 2012). In contrast, insertions and deletions of nucleotides during somatic hypermutation are particularly rare (Wilson et al. 1998; de Wildt et al. 1999). As expected, most of hypermutations accumulate in the complementarity determining regions (CDRs; see fig. 1), which are directly involved in binding to the antigen. This is partially due to the higher presence of mutational hot-spot sequences in these regions (Wagner et al. 1995; Cowell et al. 1999), and partially due to their plasticity. In fact, CDRs constitute structurally flexible loops, and therefore can accept a wider range of mutations compared with framework regions (FRs), which have mainly a scaffolding role and must keep an ordered β-barrel structure. Remarkably, different from other genes and cell types, the hypermutation rate of antibody genes is about 10^5^- to 10^6^-fold higher compared with mutation rates in the rest of the genome (Oprea 1999), and it results in 1–2 mutations per cell generation in the most variable part of the VH and VL regions.

The availability of large antibody sequence data sets retrieved from an organism, preferably at different time points postinfection, allows to reconstruct the most likely somatic hypermutation pathway and to map the mutations that have led to an increase in antigen-binding affinities, without the need of resource consuming experimental lab work. Nevertheless, the uniqueness of this somatic evolution process requires the development of bioinformatics tools tailored for antibody sequences.

Stochastic models that describe somatic hypermutation process constitute the first building block required for the development of accurate and efficient computational methodology capable to predict antibody properties from sequence data. As somatic hypermutation involves fast mutation of antibody sequences, this process can be described using a Markov substitution model of molecular evolution. There exist a number of amino acid substitution models, such as the popular WAG (Whelan and Goldman 2001) and LG matrices (Le and Gascuel 2008), which are regularly used for modeling protein evolution. However, these models were estimated from large databases which comprised a variety of different proteins and from species ranging between prokaryotes and eukaryotes. These models therefore represent a general description of protein evolution and cannot accurately reflect the intricacies of antibody somatic hypermutation. Thus, using these general models for bioinformatics analyses of antibody sequences can lead to misleading results causing the loss of accuracy and signal during statistical inferences. For example, Gil et al. (2013) observed that using different amino acid and codon models can lead to very different inferred phylogenies. For antibody sequences, we have also observed that using LG instead of our new antibody-specific model AB often yields different tree topologies.

Indeed, numerous data-specific amino acid models have been derived, including models for mitochondrial and chloroplast proteins, as well as HIV and flu-specific models (Adachi and Hasegawa 1996; Yang et al. 1998; Adachi et al. 2000). These models have been successfully used for specific types of proteins (Martin et al. 1998; Wyckoff et al. 2000; Nishihara et al. 2006) demonstrating the need and the potential of data-specific models for proteins evolving in a distinct manner.

Currently, there is no specific model describing somatic hypermutation during the maturation of expressed antibody sequences. Moreover, only few bioinformatics studies have appeared so far (Clark et al 2006; Barak et al 2008; Zhua et al 2013); despite the importance of these seminal works, none of them provides a quantitative data-driven substitution model which can be used in well-established tree-reconstruction and sequence alignment methods. In particular, Clark et al. (2006) simply count the observed number of mutations between the germline and the rearranged sequences. For the first time, Barak et al. in 2008 suggested reconstructing parsimony-like tree structures not requiring any substitution model. More recently, Zhu et al. (2013) analyzed antibody sequence data by building a phylogeny under the assumption of molecular clock (constant rate over time) and assuming the nucleotide-based substitution model HKY (Hasegawa et al. 1985), with transition/transversion rate and base frequencies fixed by default in the utilized software rather than driven by data or the specifics of antibody evolution. Thus, due to the lack of antibody specific models, most of the immunology research groups are still using general amino acid substitution models or have created their own “in house” tools, which are usually poorly documented and lack common standards.

Here, we propose to utilize the wealth of the most recent theoretic advances in the field of computational molecular evolution (Anisimova et al. 2013) to infer an antibody-specific model (the AB model) from a large collection of publically available antibody sequence data, including recent sequence data obtained through NGS (Liao et al. 2013; Doria-Rose et al. 2014; and NCBI Sequence-Read Archives accession number ERR412888 (Menzel et al. 2014) available from http://www.ncbi.nlm.nih.gov/sra). We describe antibody sequence hypermutation by a Markov amino acid substitution process. For illustration, we discuss below some examples of practical applications, which could benefit from the use of our newly inferred antibody-specific model: 1) The detection of new antibodies with specific functionality, for example, broadly neutralizing antibodies against HIV (Wu et al. 2011); 2) in silico rematching of heavy and light chain sequences derived from bulk lysis of B cell populations and sequenced by high-throughput NGS techniques (Zhu et al. 2013); and 3) the development of tools for inferring evolutionary characteristics of B cell populations based on distributions of phylogenetic tree shape statistics (Barak et al. 2008). The AB model is publically available and easily applicable for the inference of multiple sequence alignments (MSAs) and phylogenetic trees.

## New Approach

Here for the first time, we present an antibody-specific model, denoted AB, describing the amino acid replacements in maturating antibodies. We show that for antibody sequence data the AB model largely outperforms existing general amino acid models. For this task, a large amount of antibody sequence data were assembled and classified into homogeneous (gapless) MSAs. Data for each MSA were selected using a methodology customized specifically for antibody sequences. The AB model was estimated jointly from these resulting alignments and consequently tested with large data sets. Here we provide a brief overview of our methodology, with further details available in the Data and Methods section.

In this article “antibody sequence” typically refers to the VH and VL regions of rearranged antibody sequences, unless specified otherwise. CDRs and FRs are annotated according to the IMGT numeration system (Lefranc et al. 2003).

### Creating Antibody Sequence Alignments

We used publically available antibody sequences from the ImMunoGeneTics information system (Lefranc et al. 2009), which result from different experimental settings and include antibodies targeting a variety of proteins. Estimation of an antibody-specific amino acid substitution model from this large variety of antibody sequences allowed to minimize the effect of biases caused by the use of specific experimental settings.

Due to extreme length variability, all data were carefully filtered and grouped into homogeneous sets. The great length variability within antibody homologous structural regions (particularly the highly variable CDR 3, ranging between 3 and 20 residues in the used data set) allows antibodies to bind to a large variety of antigens but at the same time makes the alignment of antibody sequences difficult. Different annotation schemes have been suggested for numbering the residues in antibody sequences (e.g., Kabat et al. 1992; Honegger and Plückthun 2001); however, these numbering schemes are of little use in indel-rich regions when it is necessary to identify homologous residues. As a consequence, when constructing alignments of such regions, a large number of gaps are usually necessary in order to compensate for the length differences between the sequences, which however does not resolve the ambiguities about which residues are homologous. Relying on alignments with gap-rich regions to estimate the antibody-specific model can have adverse effects on the accuracy of the parameter estimates.

To circumvent these difficulties, we have relied on the specifics of the antibody evolution: The length variability mostly occurs due to the insertion/deletion of nucleotides at the joining parts between the V, (D), and J gene segments in the initial rearrangement process, in addition to the use of germline gene segments possessing different lengths. We sorted antibody sequences into homogeneous sets, that is, clustering sequences originating from similar V(D)J rearrangements, with V, (D), and J gene segments of the same length and the same number of indels at their joining parts. This allowed us to create MSAs for each resulting homogeneous set of antibody sequences with the same length in each of the constituent FRs 1–4 and CDRs 1–3. As a result, the majority of sequences in each MSA was derived from a small subset of V, (D), and J germlines, so that the phylogenetic signal was dominated by somatic hypermutation. The germline gene sequences themselves were not included into the alignments. Checking these resulting gapless MSAs, we confirmed that by virtue of our sorting algorithm the FRs were aligned unambiguously suggesting character homology within the CDRs without the need to introduce any gaps (see Data and Methods for details). Using this approach, we transformed the total of 23,081 sequences and 1,759,389 residues into 224 gapless alignments of highly homologous antibody sequences (antibody MSAs).

These MSAs were subsequently used to study mutational patterns at homologous residues: 213 randomly selected MSAs were used as a training set, *D*_tr_, for estimating the new AB model, whereas the remaining 11 MSAs were used as a test set, *D*_test_, to test the model’s performance. In addition, large NGS data, *D*_NGS_, from immunized mice and from HIV^+^ human donors taken at different time points postinfection were used to confirm the performance of the AB model through statistical testing. The antibody sequence alignments are provided for download in the Supplementary Material online.

### The AB Model for Antibody Maturation Process

In any given alignment of antibody sequences, the mutational history of antibody maturation was modeled independently for each homologous residue as a stochastic Markov process of amino acid substitutions along a tree structure (or genealogy) describing the history of sampled sequences as they evolved from their respective germline sequences. The MSAs of homogeneous antibody sets were used to estimate an empirical antibody-specific amino acid substitution model, defined by a 20 × 20 generator matrix *Q*_AB_* = *{*q_ij_*} of instantaneous rates of replacement between amino acids. Following common practice (to avoid overparameterization), time homogeneity and reversibility were assumed, so that each instantaneous rate of change was decomposed as a product *q_ij_* = *s_ij_ π_j_* for any *i* ≠ *j*, where *s_ij_* is a symmetric amino acid exchangeability rate and *π_j_* is a stationary frequency of targetamino acid (Yang 2006). The transition probability matrix *P*_AB_ (*t*)* = *exp (*t Q*_AB_) allows to compute probabilities of amino acid changes over a given time interval. This can be used to compute the likelihood function, which is the overall probability of observing a set of MSAs given a substitution model *Q*_AB_ and a set of trees relating the antibody sequences for each MSA. Parameters of the model are then estimated using the maximum likelihood (ML) approach, that is, by optimizing the joint log-likelihood function computed over all the MSAs in *D*_tr_.

### The Estimation of Maturation History and Model Parameters

**Fig. 2.**
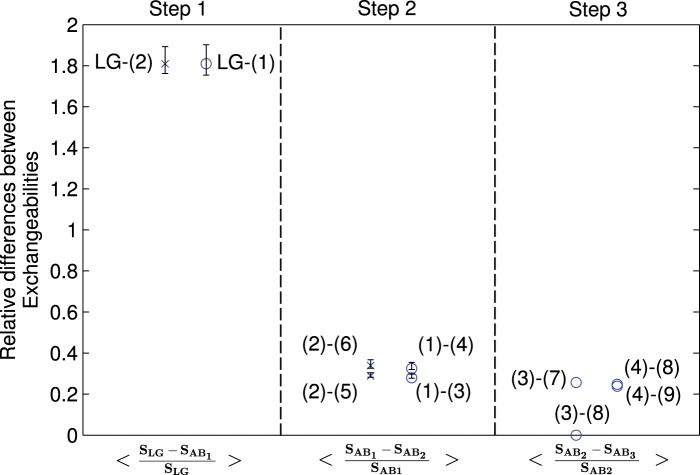
The learning progress in the estimation of the AB model is supported by the decrease in relative differences between the exchangeability values at the more advanced learning steps (see table 1 for the summary of the iterative procedure). The three sections of the graph reflect the use of different initialization parameters for XRate (Klosterman et al. 2006) and PhyML (Guindon et al. 2010): The first section shows the difference to LG parameters after the first learning step (starting the learning algorithm with the LG model parameters), the second section shows the difference for the new models (3)–(6) relative to models 1 and 2 obtained in the first step. Finally, the third section shows differences for models obtained in the third iteration step. The numbers in brackets refer to the model numbers as defined in table 1. The error bars show the standard deviations of the relative differences between different learning steps obtained by bootstrapping the set of antibody MSAs for each following learning step. They are centered about the average values of the exchangeabilities obtained from the bootstrapped alignments.

To estimate the parameters of the antibody substitution matrix, we use the expectation maximization (EM) algorithm and an iterative multistep learning approach (cf. Data and Methods). Briefly, for each MSA a genealogy was inferred using the best available amino acid model with site-rate variation and used for the computation of the joint likelihood function over all MSAs in *D*_tr_. Next, a new amino acid model was estimated for *D*_tr_. This procedure was repeated, each time with different initial values, using newly obtained estimates from the previous round (for both exchangeability rates and optionally also amino acid frequencies). We halted the procedure as soon as the joint likelihood ceased to increase. As a result, 12 candidate amino acid matrices were estimated at different stages of the procedure and were subsequently evaluated in terms of the differences between their optimized log-likelihoods (table 1) and the exchangeability rate estimates (fig. 2). This allowed us to monitor whether the matrices estimated at different stages of the procedure were converging to the same peak or a plurality of peaks on the likelihood surface.

**Table 1.** Iterative Estimation of Candidate Models for Antibody Sequences.

Existing/Inferred Model	Model Used for Tree Inference	Initial *s_ij_*	Initial π*_i_*	Log-Likelihood per Site
WAG	WAG	—	—	−45.396
LG	LG	—	—	−45.976
1	LG	LG	π_MSA_	−44.257
2	LG	LG	π_LG_	−44.231
3	1	1	π_MSA_	−44.239
4	1	1	${\overset{⌢}{\pi }}_{1}$	−44.237
5	2	2	π_MSA_	−44.246
6	2	2	${\overset{⌢}{\pi }}_{2}$	−44.263
7	3	3	π_MSA_	−44.273
8	3	3	${\overset{⌢}{\pi }}_{3}$	−44.239
9	4	4	π_MSA_	−44.257
10	4	4	${\overset{⌢}{\pi }}_{4}$	−44.247
11	WAG	WAG	π_MSA_	−44.237
12	WAG	WAG	π_WAG_	−44.249

### Validation and Interpretation of the AB Model

To ensure the accuracy and sensitivity of our estimation procedure, we used two different data sets to evaluate and confirm the performance of the AB model. The first selection of the three best models was performed on *D*_test_ composed from one or several MSAs for each of human antibody heavy chain, κ-light chain, λ-light chain, mouse antibody heavy chain, and κ-light chain. These data were sufficient to evaluate whether any one of the 12 models fitted *D*_test_ data significantly better than existing (general) amino acid substitution models; however, this sample was insufficient to select the best model with certainty.

Thus, we have used the additional *D*_NGS_ data consisting of 150 MSAs with 7.1 × 10^6^ residues. These alignments comprised sequences similar to those in *D*_test_ (human heavy, human κ-/λ-light chain, and mouse heavy chain sequences). Wilcoxon signed-rank test was used to determine the best model describing the somatic evolution for this set of antibody sequences.

For the biological interpretation of the best fitting model AB, we used the modularity maximization approach that is widely applied in network science (Newman 2004). To do so, we considered the exchangeability values in the AB model as weights (connection strengths) in a network with 20 amino acids (nodes). We inferred an optimal number of amino acid clusters so as to maximize the sum of the connection strengths (exchangeability values) within each cluster and to minimize the strengths of the connections transgressing cluster borders (cf. Data and Methods for mathematical details). Based on the inferred modularity scores and physicochemical properties of amino acids (Taylor 1986; Betts and Russell 2007) in inferred clusters, we were able to compare the substitution properties of the AB model and the general LG model.

## Results and Discussion

Using large sequence data from different species and antibody classes, we inferred 12 candidate models describing antibody somatic evolution among which one best-fitting model was selected for its better description of antibody sequence data. We refer to this model as the AB model.

**Fig. 3.**
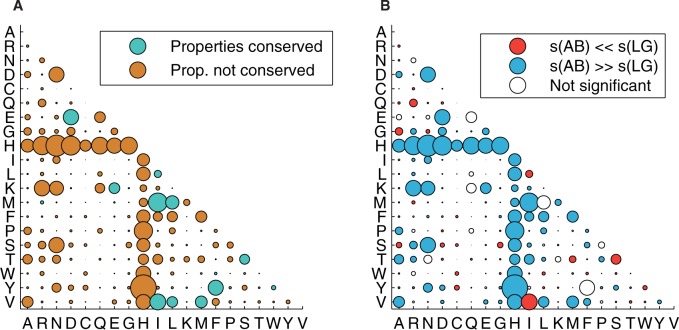
The AB model for somatic hypermutation: (*A*) The amino acid exchangeability matrix: The area of each bubble represents the exchangeability *s_ij_* between amino acids *i* and *j*. Blue color of bubbles indicates that the main physicochemical properties of the amino acids are conserved after a change (as defined by Taylor 1986 and revised by Betts and Russell 2007). Brown color indicates that at least one of the main amino acid properties changes. (*B*)A comparison of the exchangeability matrices for AB and LG models: Red and blue bubbles show pairs of amino acids for which exchangeability rates are significantly different between the LG and AB models. In total, 100 bootstrapped alignments of the antibody training MSAs have been used to obtain distributions of the exchangeability rates in the AB model. The values are declared statistically different if the exchangeability value of the LG model exceeds the 0.975 or falls below the 0.025 quantile of the bootstrap distribution of the corresponding AB exchangeability rate.

**Fig. 4.**
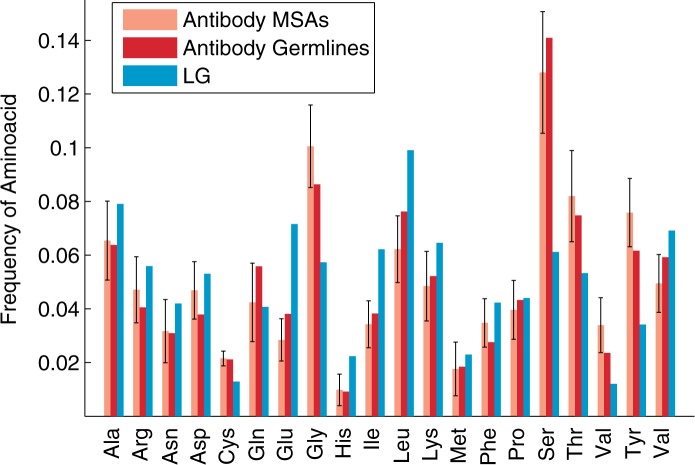
The amino acid frequency distributions for the LG model (Le and Gascuel 2008) compared with the antibody-specific distributions that are inferred 1) from the human and mouse antibody MSAs in the training set, and 2) from the germline sequences downloaded from the IMGT database (Lefranc et al. 2009). The error bars show the standard deviations in the amino acid frequencies between different homogeneous (gapless) MSAs constructed from the IMGT data for mouse and human (heavy chain and light chain). The amino acid distribution inferred from the human and mouse antibody MSAs in the test set is recommended for use with the AB model of the antibody maturation process.

The estimates of the AB exchangeability rates and stationary amino acid frequencies are shown in figures 3 and 4, respectively.

Below we demonstrate that the AB model outperforms general amino acid substitution models such as LG and WAG, as assessed by the gains in the optimized log-likelihood values when applied to antibody sequence data. This suggests that the AB model better reflects the biological processes involved in the somatic hypermutation and selection steps that lead to the in vivo maturation of antibody sequences in response to an antigen. Thus, the AB model should be used for bioinformatics analyses of antibody sequences. Below we discuss the fit of the new model, the robustness of model estimates, its comparison with existing models, and the biological interpretation of the observed findings.

### The AB Model Provides Better Fit to Antibody Sequence Data

**Fig. 5.**
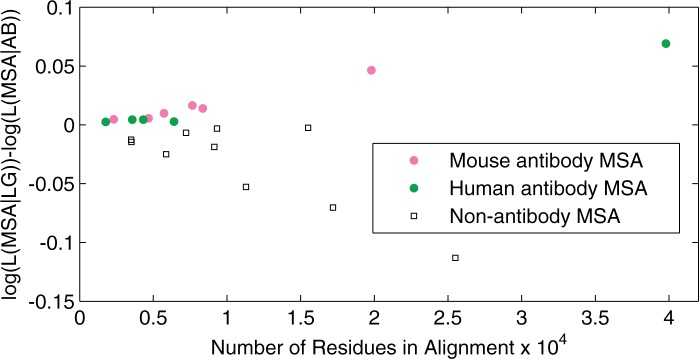
The fit to data of the AB model compared with the general model LG (Le and Gascuel 2008). Differences in log-likelihoods per site are shown for 11 homogeneous (gapless) antibody MSAs from the IMGT database and for 11 nonantibody MSAs from TreeBase (Sanderson et al. 1994). Log-likelihood values are optimized under models AB+Γ+I+ F_AB_ and LG+Γ+I+ F_AB_.

Invariably, we observed that the AB model provided a better fit to antibody sequence data compared with the general model LG on both testing data sets (table 1 and fig. 5). For example, on *D*_test_ the AB+Γ+I+F model (invariant sites “+I” with four Γ-rate categories “+Γ” and amino acid frequencies inferred from each MSA “+F”) showed a substantial log-likelihood gain: On average, 1.78 units per site compared with LG+Γ+I+F and 1.13 units per site compared with the WAG+Γ+I+F model. When using the antibody-specific stationary distribution “+F_AB_” — inferred as model parameters from *D*_tr_ — the difference between AB+Γ+I+F_AB_ and LG+Γ+I+F_AB_ was 1.74 log-likelihood units per site, whereas between AB+Γ+I+F_AB_ and WAG+Γ+I+F_AB_ this difference was 1.16. The observed differences in optimized log-likelihood values were so large that even for this limited number of 11 MSAs in *D*_test_ we could confirm that AB significantly outperforms LG and WAG (Wilcoxon signed-rank test, *P* value < 0.01).

However, we do not expect the AB model to provide a good fit for typical nonantibody proteins compared with the existing general models. To verify this, we randomly selected 11 MSAs of regular proteins from the TreeBase (Sanderson et al. 1994) used to infer the LG model. Indeed, for this set, the AB model did not provide better fit. In fact, when the LG model frequencies were used (+F_LG_), the per site log-likelihood decreased by 0.72 for the AB+Γ+I+F_LG_ model relative to LG+Γ+I. This indicates that the AB model indeed captures the specifics of the mutation-selection processes involved in somatic hypermutation during the maturation of antibody sequences. Moreover, the improved model fit is not only due to differences in stationary amino acid frequencies but also due to exchangeability rates.

It is interesting to note that among the general amino acid models, WAG provided on average a better fit for antibody alignments in *D*_test_, outperforming LG by 0.58 units per site. This shows that the advantage of LG over WAG cannot be generalized to all protein types, with antibody sequences being one example.

### Convergence and Reliability of the AB Model Estimates

During the iterative estimation procedure, we have repeated the learning algorithm with different initial values, gradually improving the model until no further improvement in the log-likelihood values for the test alignments could be obtained. We monitored the convergence of the model estimates by assessing the mean absolute value of the relative differences between the estimates of exchangeability rates {*s_ij_*} and {s_ij_*} from two consequent iterations, computed as Δ*_ij_* = |(*s_ij_* − s_ij_*)/*s_ij_*| for any pair of distinct amino acids *i* and *j*, whereby {s_ij_*} are the current estimates obtained by using the previous estimates {*s_ij_*} as starting values (see Data and Methods for details of the iteration procedure). Indeed, we observed a large 1.8-fold decrease of the Δ*_ij_* values in the first learning step of the iteration procedure, followed by 0.29–0.34 fold increase in the second learning step and 0.24–0.25 in the third learning step. For one set of parameters, learning of model (3) after the second learning step failed to alter the exchangeability matrix (fig. 2). The overall trend supports the convergence of the learning algorithm.

Next, we examined the statistical confidence of the estimated AB exchangeability rates using bootstrap resampling of the MSAs in *D*_tr_*.* Figure 2 shows the bootstrapped values of Δ*_ij_*_,_ where bars represent standard deviations of Δ*_ij_* in the bootstrapped alignments. This analysis allowed to assess the sensitivity of the model estimates to the presence of specific sites within the original alignment, as bootstrapped distributions are similar to generating MSAs by increasing the weights for certain functionally diverse sites compared with the original *D*_tr_. For all pairs of amino acids, the distribution of the exchangeability rate estimates in the bootstrapped set was roughly symmetric and had a single peak within 0.5 SD from the estimated exchangeability value for the original set of MSAs. The same was true for the relative differences Δ*_ij_*, indicative of the robustness of the learning approach and the reliability of the obtained estimates.

The different initialization values and stationary amino acid distributions used in the learning process yielded a total number of 12 candidate models (as detailed in table 1). All the exchangeability matrices were highly correlated (*r* > 0.97 between any of models (1)–(12)). In contrast, the correlation of the exchangeabilities between candidate model (4) and LG was only 0.70. For *D*_test_ data, the largest log-likelihood difference per site between any of these models was 0.04, which is very small compared with the difference between any of (1)–(12) models and LG (cf. table 1). The high correlation between estimates from the candidate AB models and their very similar optimized log-likelihoods suggested that models (1)–(12) are in close proximity on the likelihood surface.

The sample size of *D*_test_ was not sufficient to evaluate if best fitting model (2) yielded statistically significantly higher likelihoods compared with other candidate models (Wilcoxon signed-rank test was not significant). Thus, we have used the additional NGS data to perform the Wilcoxon signed-rank test for the top three models (2), (3) and (4) from the 12 candidate models. Applied to the *D*_NGS_ data, model (4) provided significantly better fit compared with any other substitution model (*P* value << 0.01).

We have thus selected model (4) as the final antibody-specific substitution model AB that provides the best description of mutational patterns during somatic hypermutation in antibody sequences.

We have further compared the likelihood of different models with different possible amino acid frequencies. The empirical amino acid frequencies estimated from *D*_tr_ (rather than from an MSA at hand) provided the best fit for most antibody MSAs. This distribution is shown in figure 4.

### Differences from Existing General Amino Acid Substitution Models

#### Stationary Amino Acid Frequency Distribution

The antibody sequences in the training set exhibited higher frequencies of Cys, Gly, Ser, Thr, Val, and Tyr, but lower frequencies of His, Ile, Leu, and Lys (fig. 4) compared with the LG model. All amino acid frequencies in germline V, D, and J gene segments for human and mouse were within 1.1 SD from their correspondent mean frequencies in *D*_tr_.

#### Exchangeability Matrix

The AB exchangeability rate matrix is depicted in figure 3*A* and *B*. For several amino acid pairs, the estimated exchangeabilities were significantly different from the LG values. We used the bootstrapped alignments and considered the exchangeability values outside the 2.5–97.5% quantiles to be significantly different form the LG values (fig. 3*B*). Notably, the high exchangeabilities between His and many other amino acids are counterbalanced by His’s lowest stationary frequency, which largely decreases the flow between His and any other amino acid in the Markov model. This particularity of His may be due to its high pH-sensitivity, which leads to a change in its net-charge upon internalization (Igawa et al. 2010; Strauch et al. 2014).

#### Model’s Modularity

**Fig. 6.**
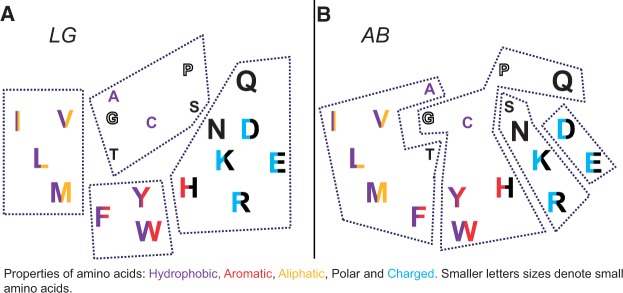
The modularity maximization analysis showing the inferred optimal clusters of most frequently exchanged amino acids: (*A*) For LG with modularity *M* = 0.38 and (*B*) for AB with modularity *M* = 0.24. The significantly higher modularity for LG shows the clearer structure of the LG model, with a tendency to conserve amino acid properties. Colors and letter size reflect the physicochemical properties of amino acids as suggested by Taylor (1986) and revised by Betts and Russell (2007).

For a further insight into the exchangeability patterns implied by the AB and LG substitution models we have used a modularity maximization approach, which allowed to assess whether clusters of frequently exchanging amino acids existed (see Data and Methods). The best partition of amino acids for models LG and AB into an optimal number of clusters is shown in figure 6*A* and *B* respectively. The maximized modularity values were 0.38 for the LG matrix and 0.24 for the AB matrix. This difference is significant, as the distribution of optimized modularity values for the estimates of the AB models inferred from bootstrapped replicates had the standard deviation of only 0.01. Modularity values greater than 0.3 are considered representative of clear partitions of nodes within a network into separate modules (Newman 2004). The LG exchangeability matrix can thus be considered as providing clear partitions of amino acids, whereas the AB exchangeability model does not suggest such clustering.

**Fig. 7.**
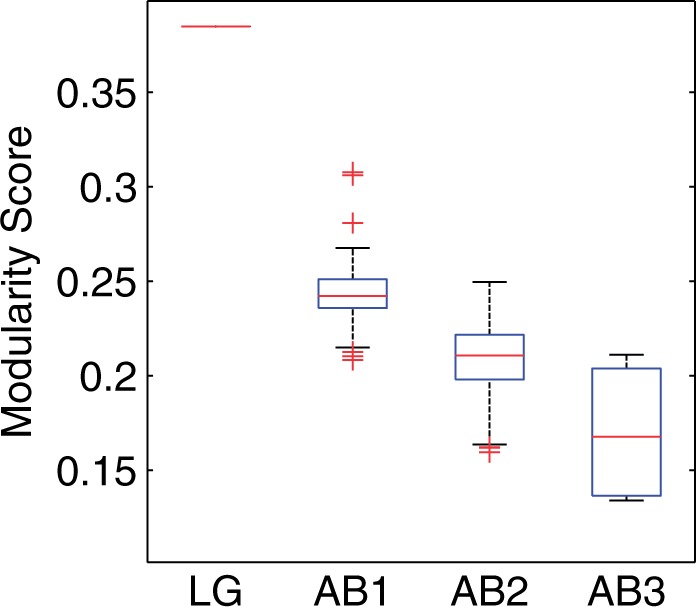
Modularity values of the amino acid networks implied by the LG model and the candidate antibody-specific models at different learning steps are shown (details cf. table 1). AB1 refers to the modularity distribution of the antibody exchangeability models inferred after the first learning step, which results in estimated models (1) and (2) together with their bootstrap distributions, and AB2 refers to models (3)–(6) together with their bootstrap distributions. The final third step is represented by AB3 and includes models (7)–(10). This shows that the modularity values decreased at each learning step.

Interestingly, at each new learning step of our estimation procedure, we observed decreasing modularity property of the estimated AB exchangeability matrices (whereas the log-likelihood was still increasing at the first and second optimization steps). Figure 7 shows the differences in modularity values at the different learning steps of the model. This supports our observation that the decreased modularity property for the AB matrix must be reflecting the specifics of antibody sequence evolution.

### Biological Interpretation of the AB Model

Several ways have been proposed on how to classify amino acids according to their physicochemical properties; for example, Taylor (1986) suggested a classification based on properties such as hydrophobicity, size, charge, and other specific side chain peculiarities (fig. 6*A* and *B*). Le and Gascuel (2008) demonstrated that the majority of observed amino acid exchanges in the LG model occurred between pairs of amino acids having similar physicochemical properties (high *s_ij_* for exchanges Ile ↔ Val, Phe ↔ Tyr, Lys ↔ Arg, Asp ↔ Glu, etc.). The application of the modularity maximization algorithm to the LG exchangeability matrix allows the inference of groups within which amino acids are preferably exchanged. The inferred modules are presented in figure 6*A* for the LG model and match well the aforementioned amino acid classification.

The application of the same modularity maximization algorithm to the AB model yielded different results (fig. 6*B*): The amino acid network exhibited no clear modularity structure (modularity less than 0.3) and the suggested (weak) modules do not reflect the amino acids physicochemical properties. For example, small amino acids such as Gly and Cys are clustered together with large aromatic amino acids. This suggests that the processes involved in the somatic antibody evolution are largely different from those shaping the evolution of other proteins. The LG model is characteristic of conservation at the protein level during evolution, which either prevents amino acid changes or selects for amino acids having similar physicochemical properties that are likely to have a lower impact on the function of the protein.

Somatic mutations in antibody sequences, however, eventually lead to an increase of binding affinity to the antigen and to the improved complementarity between the heavy and light chain protein interface, which is unlikely to be achieved under negative selection on the protein level. Instead, a diversifying mechanism is required here. Yaari et al. (2012) suggest that the evolution of the antibody CDRs is dominated by positive selection whereas the FRs’ somatic evolution is dominated by negative selection. As the phylogenetic signal from somatic antibody evolution is largely dominated by mutations in the CDRs (FRs have less mutations), the evolution of complete variable parts of antibodies should be dominated by positive diversifying selection. This is in agreement to the low modularity of the AB model and the observed dominance of property-altering amino acid exchanges in the antibody MSAs (fig. 3*A* and 6*B*).

### Usage and Availability

The AB model for phylogenetic inferences and alignment of antibody sequences is provided as a Supplementary Material online. In addition, this model has been implemented in the CodonPhyML program for inferring phylogenetic trees from MSAs (Gil et al. 2013). The AB model can be used in order to accurately align antibody sequences (i.e., with programs allowing for user substitution matrices like the fast implementation of probabilistic phylogeny-aware graph-based program ProGraphMSA [Szalkowski 2012; Szalkowski and Anisimova 2013]).

When using the AB matrix for bioinformatics analyses of antibodies, we suggest using the stationary amino acid distribution as estimated here for the AB model from our training set (+F_AB_ option), and not those empirically estimated from each alignment as is common practice (+F option). As the +F option involves 19 additional parameters and at the same time AB+F_AB_ outperforms the AB+F models according to our calculations, estimating the frequency distribution from individual antibody alignments is strongly discouraged. The lower log-likelihoods observed when +F option was used are probably due to stochastic effects caused by the limited-size alignments of highly similar antibody sequences (e.g., due to conserved FRs).

## Conclusion and Future Prospects

General amino acid models do not capture the sophisticated patterns of somatic hypermutation in antibodies. Therefore, here we have estimated and tested the antibody-specific model AB that describes somatic hypermutation during the antibody maturation. The follow-up analyses by modularity maximization suggest that the mutational processes in maturating antibodies are consistent with a sui generis mechanism driving the diversity of antibody sequences. The advantage of the AB model over other (general) substitution models was evident throughout the different alignments of human and mouse heavy/light chain sequences. The AB model can also be used for analyzing antibody sequences from other jawed vertebrates besides human and mice, given that they share the somatic evolution mechanism through an ancestral relationship.

In the beginning of the study different approaches have been considered, one of them was to infer separate matrices for the FRs and CDRs. Alignments consisting of either FRs or CDRs were created so as to infer separate substitution models for these regions. However, as substitutions within the FRs are rare, this approach could not provide sufficient phylogenetic signal to infer reliable statistical models for the FRs. Additionally, new evidence questions the validity of the functional separation of the antibody sequences into regions responsible for structure and binding to the antigen, so that some positions within the CDRs would never participate in antigen binding, whereas some off-CDR parts contribute critically to the binding between the antigen and the antibody (Sela-Culang et al. 2013).

In conclusion, the AB model is the first step toward the modeling of hypermutation in antibody sequences, as it provides the fundamental block for the development of bioinformatics methods that rely on the analyses of phylogenetic patterns in antibody sequences. Additional methodological research and extensive in vivo investigations are needed to find the optimal way to make use of alignments and phylogenetic trees inferred using the AB model. The rooting of inferred antibody phylogenies will need particular attention. A number of tools allow for identification of the V, (D), and J germline gene fragments for each rearranged antibody sequence (Gaeta et al. 2007; Ye et al. 2013). These gene segments can then be concatenated and used to define the rooting for antibody sequence phylogenies. Such rooted trees can then be used to accurately infer evolutionary relationships between antibody sequences.

In particular, we see a use of the AB model within the frame of the work of Wu et al. (2011) where it would provide more accurate measures of biological distances between a known broadly neutralizing antibody (such as VRC01) and newly obtained antibody sequences. It further allows identifying antibody sequences with the largest evolutionary distances from their respective germline sequences. The combination of both properties can be used to identify new broadly neutralizing antibody candidates.

In another application, independent trees for heavy and light chain antibody sequences can be constructed so as to identify pairs of matching chains which have been separated while sequencing the B cells (Zhu et al. 2013). In this case, more accurate phylogenetic trees inferred from large sets of sequences would allow to detect more heavy and light chain sequence pairs than if general amino acid models were used.

We also expect that accurate trees inferred from antibody sequences should show specific characteristics of the somatic antibody sequence evolution: The diversifying sui generis process will leave a characteristic imprint on the trees, underlying the dynamics of antibody hypermutation. Applying the AB model to large-scale sequences, one can follow B cell populations of antibodies as opposed to single clones. Insights into B cell evolution dynamics can be gleaned from statistical analyses of the tree shape distributions inferred from sets of sequences coming from different immune system organs, different species, or from organisms affected by different diseases. Such work, therefore, may lead to the development of additional tools for monitoring the progress of the immune system’s reaction to diseases.

The applications listed here are but only a few examples of how the AB model can advance immunology research. In conclusion, this work has the potential not only to provide insights into the evolution of antibody sequences for specific targets but also to open up a wide field of phylogeny-based immunology research allowing to monitor the current state of the immune system and the evolution of the humoral response to diseases.

## Data and Methods

### Assembling Biological Sequence Data for Training and Test Sets

To minimize biases toward specific animals or experimental settings (e.g., target, immunization scheme), we used antibody sequences from human and mice from the ImMunoGeneTics database (www.imgt.org; version dated January 2013). We selected species for which a sufficient amount of antibody sequences were available (more than 5,000 sequences). All types of antibody sequences were used and analyzed separately in different groups, subdivided into human heavy chain, human λ-light chain, human κ-light chain, mouse heavy chain, and mouse κ-light chain. As mouse light chain sequences are largely dominated by κ-sequences, we did not use the λ-sequences due to the low number of such sequences available. Sequences in each of these groups were aligned using MAFFT (Katoh et al. 2002; Katoh and Standley 2013) and the resulting MSAs were manually verified in Jalview (Waterhouse et al. 2009) by applying the IMGT annotation rules for antibody sequences (Lefranc et al. 2009). In detail, for each sequence, characteristic amino acid patterns were identified and their alignment was enforced in all groups. Differences in lengths were balanced by the introduction of gaps into the alignments. At this step, sequences with stop codons, ambiguities or obvious sequencing mistakes, or missing complete CDRs or FRs were discarded. Subsequently, the MSAs were realigned in MAFFT followed by another manual verification and realignment step. Many sequences lacked the beginning of the FR1 and/or the end of the FR4 regions. Consequently, about half of the FR1 and of the FR4 region had to be truncated in all sequences in order to maintain the maximum of same-length sequences in the alignment. At this stage, sequences missing more than half of the FR1 or FR4 sequences were also discarded.

As a result of the described procedure we selected 8,919 mouse heavy chain sequences, 1,080 mouse κ-light chain sequences, 8,062 human heavy chain sequences, 2,634 human κ-light chain sequences, and 2,386 human λ-light chain sequences.

To create gapless alignments we first annotated all sequences according to the lengths of their FRs and CDRs and then created MSA of only those sequences, which had the same number of residues in each fragment (FR1–FR4 and CDR1–CDR3). We required a minimum of three sequences for each single MSA.

Overall, our procedure resulted in 113 MSAs of human heavy chain and 43 MSAs for human light chain MSAs, among which 25 λ-light chain and 18 κ-light chain alignments. For the mouse data, the resulted number of MSAs was 55 for the heavy chain and 13 for the light chain sequences. The MSA size ranged from 3 to 1,308 sequences (with a mean of 83 sequences and an average length of 100 amino acids). Out of the total of 224 MSAs, we randomly selected 213 MSAs for estimating the AB substitution model (training set *D*_tr_), the remaining 11 alignments were reserved for validation purposes (test set *D*_test_) and were not used for the estimation of the AB model.

### Preparation of the Additional Test Sequence Alignments

The downloaded mouse heavy chain sequences were taken from a pool of nine mice immunized with 50 μg alum-precipitated chicken gamma globulin and sacrificed 14 days postimmunization. Further experimental details are provided at http://www.ncbi.nlm.nih.gov/sra/?term=ERR412888 (last accessed December 21, 2014).

Human heavy chain sequences and λ-light chain sequences were obtained 144 weeks post-HIV infection of a patient by Liao et al. (2013); sequence data were obtained from the NCBI Sequence Read archive; accession numbers for the heavy and light chains sequences are SRX297269 and SRX297274, respectively. Antibody sequences from another HIV^+^ patient were obtained 16 weeks postinfection from Doria-Rose et al. (2014) (accession numbers SRX398466 and SRX398467 to heavy and κ-light chain sequences, respectively).

In total, after a quality-control filter for ambiguous characters and stop codons, 136 × 10^4^ mouse and 891 × 10^3^ unique human sequences were obtained. Next, antibody MSAs were created for mouse (heavy chain) and human (heavy chain, κ-light and λ-light chain) data. Due to the vast amount of sequence data we could create antibody sequence alignments originating from similar (if not same) V(D)J rearrangements, that is, sets of sequences originating from the same set of V, (D), and J gene segments and having the same number of indels in the joining regions V–D and D–J or only between V–J gene segments for heavy and light chain sequences, respectively. To do this, we calculated a scoring matrix (Henikoff and Henikoff 1992) and used it to identify the most likely set of V, (D), and J gene segments for each antibody sequence from the NGS data by local pairwise alignment (Smith and Waterman 1981) between the respective antibody sequence and any of the possible germline gene segments. For each sequence, the most likely V, (D), and J germline gene segments were selected based on the highest alignment score, different germline gene sets were used based on the species and the sequence type. Each of the V, (D), and J gene segments was locally aligned only within a region of their expected position within the antibody sequences so as to save computational time and to avoid misleading alignments. Sequences having the same closest V, (D), and J germline gene segments, best aligning at the same position, and having the same length in each of their FR1–4 and CDR1–3 were combined into individual MSAs. If the size of the MSA exceeded 850 sequences, it was split into smaller alignments. MSAs containing less than 175 sequences were discarded. This procedure resulted in a large number of gapless MSAs of antibody sequences out of which we have randomly selected 55 MSAs from mouse antibody sequences and 95 MSAs from two different HIV^+^ patients. The final *D*_NGS_ data set contained 150 MSAs with a total of 7.1 million residues.

### The Iteration Procedure for the Estimation of the AB Model

The overall goal of the learning algorithm was to maximize the likelihood of the training set *D*_tr_ by learning the set of the substitution matrix parameters *Q*, and the set of all branch lengths *T* for genealogies {*T_i_*} corresponding to MSAs {*D_i_*} in the training set *D*_tr_:

$$\log L(Q,T;D_{tr})=\log\Pi_{i}L(Q,T;D_{i},T_{i})$$

*Q* and *T* were estimated by maximizing the joint log-likelihood function through a multistep EM algorithm (Holmes and Rubin 2002)*.* The procedure comprised several learning steps (iterations). At each step, genealogies *T_i_* for individual MSAs were inferred with PhyML v.3.0 (Guindon et al. 2010) using the best-known general amino acid model at the time of the iteration process (LG at step 1), always starting the heuristic search with a maximum parsimony tree, four discrete categories of Γ-distributed rates to account for site-rate heterogeneity (Yang 1996), and invariant sites (+I). The α shape parameter and the proportion of invariable sites π_invar_ were estimated from each alignment independently. Using the Γ-rate model with constant sites allowed to account for the evolutionary rate differences in different regions (FR and CDR) of antibody sequences.

As the amino acid frequency distribution in our training set deviated from that of the LG model (see fig. 4), we always used empirical frequencies π_MSA_ estimated from data *D*_tr_ to infer the phylogenetic trees. This was computed over all MSAs in the training sets (rather than taking empirical frequencies from individual MSAs) as this strategy gave higher likelihood gains per site and also was more robust to stochastic errors when inferring the initial frequency distributions from small MSAs.

At each iteration step, the EM procedure was performed using XRate (Klosterman et al. 2006) over the set of MSAs and the inferred genealogies. Just like when inferring genealogies, all EM runs were performed assuming the best amino acid model as known at the time of the iteration (summarized in table 1). During the first iteration, both the genealogy inference and the EM routine were performed assuming the most recent general amino acid model LG (Le and Gascuel 2008) and different initial amino acid frequencies π_MSA_ and π_LG_, respectively. At the end of each EM run, the resulting estimated model included a set of ML estimates of stationary frequencies, denoted $\overset{⌢}{\pi }$, and a substitution matrix, which was transformed into an exchangeability matrix using $S_{ij}=\frac{Q_{ij}/\pi_{j}+Q_{ji}/\pi_{i}}{2}$ under the assumption of the detailed balance condition of the Markov process. The latter was done even though the reversibility was enforced by XRate: Due to numerical effects, the summands $Q_{ij}/\pi_{j}\text{and}Q_{ji}/\pi_{i}$ were not identical although very close. To create comparable exchangeability matrices, the substitution rate matrices were normalized to $\Sigma_{i,j,j=i}s_{ij}\pi_{i}\pi_{i}=1$ with $\pi_{i}$ and $\pi_{j}$ taken from π_MSA_. Table 1 is showing in more detail the iteration procedure: After the first iteration, we obtained models (1) and (2) by initialing XRate with different initial amino acid stationary distributions, either π_MSA_ or π_LG_. In the next step, we used inferred genealogies with model (1) or (2) assuming π_MSA_. As outlined above, replacing π_MSA_ by the stationary amino acid distributions inferred from individual MSAs did not increase the likelihoods of either *D*_tr_ or *D*_test._ Using these recalculated genealogies, models (3)–(6) were estimated in the second learning step. For the third learning step not all combinations were computed, as they were a priori suboptimal: Models (5) and (6) had lower log-likelihood compared with models (3) and (4). Therefore for the third learning step, only models (3) and (4) were used to initialize PhyML and XRate. This resulted in four new models (7)–(10). No further improvement for model (3) could be achieved, so model (8) was identical to model (3).

For comparison purposes, we also recomputed the first step of the iteration using the general amino acid model WAG (Whelan and Goldman 2001), resulting in the estimation of new candidate models (11) and (12). Further iterations were not performed as model (2) obtained after one learning step starting from the LG model yielded a higher likelihood for *D*_test_. Additional likelihood estimations using the *D*_NGS_ confirmed that the likelihood difference between models (2) and (12) in favor of model (2) was significant using the Wilcoxon signed-rank test (*P* value < 0.01).

To summarize, the estimation procedure has resulted in 12 candidate models for describing the antibody sequences, which served as the first phase of model selection (table 1).

On *D*_test_, the three best-fitting (initialized by LG) models were (2)–(4). We tested these three models on the large NGS data *D*_NGS_ to determine the best one (table 2). The observed differences in log-likelihood per site (log-lh/site) between heavy chain and light chain sequences are likely due to the larger number of polymorphic sites and the larger alignment sizes of the human light chain MSAs. Model (2) slightly outperformed model (4) for mouse sequences by 0.01 log-likelihood units. For any human antibody sequence alignment, model (4) yielded the highest log-likelihood values. The complete set of 150 alignments in *D*_NGS_ has shown the highest log-lh/site values for model (4). Additionally, we have performed Wilcoxon signed-rank test, which has confirmed that model (4) significantly outperforms models (2) and (3), *P* value << 0.01. Model (4) was thus chosen as the AB model.

**Table 2.** Optimized Log-Likelihoods per Site for Three Best Fitting Candidate Models on NGS Data.

Alignment in *D*_NGS_	No. of MSAs	Mean No. of Residues per MSA	Log-lh per Site, Model 2	Log-lh per Site, Model 3	Log-lh per Site, Model 4
Mouse HC	54	44.8k	−38.86	−39.18	−38.87
Human HC	46	33.9k	−36.31	−36.26	−36.23
Human KC	24	62.6k	−125.50	−125.41	−125.38
Human LC	25	62.8k	−116.60	−116.52	−116.48
All types	150	47.3k	−60.12	−60.21	−60.07

The statistical distribution of the exchangeability coefficients of the AB model was assessed using bootstrapping. For each MSA in the training set, 100 bootstrap replicate MSAs were created by drawing (with replacement) columns from the MSA. For each of 100 sets of bootstrapped MSAs, the model was re-estimated using the procedure as shown in table 1, resulting in 100 bootstrapped exchangeability matrices for the models (1)–(6). These data were used to calculate the distribution of the modularity values at different learning steps (fig. 7) and to evaluate whether the AB exchangeability rates were significantly different from those of the LG model (fig. 3*B*).

### Network Modularity Applied to the AB Exchangeability Matrix

Modularity measures can be used to access the structure of a given network, such as the presence of clusters (Newman and Girvan 2004). Here, we use the extension of the modularity measure to weighted networks (Newman 2004). The rationale for the modularity measure is the following: A good partition of a network into modules must comprise many within-module links and as few as possible between-module links (Guimera and Amaral 2005).

We apply the modularity maximization to the AB and the LG exchangeability matrices to better understand their structures. Each exchangeability matrix defines a symmetric weighted network consisting of 20 nodes representing the amino acids, with connection strengths represented by the exchangeability rates between the respective amino acids. No further normalization of the exchangeability matrix is required, as multiplication of exchangeability rates with a constant does not affect the modularity score or modularity structure.

Formally, given a matrix with exchangeabilities for each pair of amino acids its modularity can be defined as follows:

$$M=\sum_{s=1}^{r}\left[\frac{l_{s}}{L}-{\left(\frac{d_{s}}{2L}\right)}^{2}\right],$$

where *r* is the number of clusters, *l_s_* is the sum of the connection strengths (exchangeabilities) having both ends within cluster *s*, *d_s_* is the sum of all exchangeabilities with at least one end in cluster *s*, and *L* is the sum of all exchangeabilities. To find the optimal amino acid partition structure defined by a model, we performed the network modularity maximization using an algorithm similar to the one used by Guimera and Amaral (2005).

The range of *M* is between –0.5 and + 1, where *M* = 0 signifies the lack of any network structure indicative of a random clustering of nodes in a network. *M* > 0.3 indicates a clear division of the nodes into clusters (Newman 2004). Negative values relate to modularity structures having fewer within-cluster links than one would expect by random chance. The modularity value stays unaltered if all connection strengths are multiplied by a constant, which is convenient as exchangeability matrices can be multiplied by any constant without the model being changed.

## Supplementary Material

Supplementary material is available at *Molecular Biology and Evolution* online (http://www.mbe.oxfordjournals.org/).

Supplementary Data
