# Supplementary material for: Antibody-Specific Model of Amino Acid Substitution for Immunological Inferences from Alignments of Antibody Sequences
Source: Mol Biol Evol. 2014 Dec 21;32(3):806–19. doi: 10.1093/molbev/msu340 (PMC4327158; doi:10.1093/molbev/msu340)
Supplement: Supplementary Data [file supp_32_3_806__index.html]

Antibody-Specific Model of Amino Acid Substitution for Immunological Inferences from Alignments of Antibody Sequences — Antibody-Specific Model of Amino Acid Substitution for Immunological Inferences from Alignments of Antibody Sequences — Supplementary Data 

# Antibody-Specific Model of Amino Acid Substitution for Immunological Inferences from Alignments of Antibody Sequences

## Supplementary Data

files

**Files in this Data Supplement:**

- Supplementary Data - zip file
